# Polymorphism in the Tyrosine Hydroxylase (TH) Gene Is Associated with Activity-Impulsivity in German Shepherd Dogs

**DOI:** 10.1371/journal.pone.0030271

**Published:** 2012-01-17

**Authors:** Enikő Kubinyi, Judit Vas, Krisztina Hejjas, Zsolt Ronai, Ildikó Brúder, Borbála Turcsán, Maria Sasvari-Szekely, Ádám Miklósi

**Affiliations:** 1 Department of Ethology, Eötvös Loránd University, Budapest, Hungary; 2 Department of Medical Chemistry, Molecular Biology and Pathobiochemistry, Semmelweis University, Budapest, Hungary; The Mental Health Research Institute of Victoria, The University of Melbourne, Australia

## Abstract

We investigated the association between repeat polymorphism in intron 4 of the tyrosine hydroxylase (TH) gene and two personality traits, activity-impulsivity and inattention, in German Shepherd Dogs. The behaviour of 104 dogs was characterized by two instruments: (1) the previously validated Dog-Attention Deficit Hyperactivity Disorder Rating Scale (Dog-ADHD RS) filled in by the dog owners and (2) the newly developed Activity-impulsivity Behavioural Scale (AIBS) containing four subtests, scored by the experimenters. Internal consistency, inter-observer reliability, test-retest reliability and convergent validity were demonstrated for AIBS.

Dogs possessing at least one short allele were proved to be more active-impulsive by both instruments, compared to dogs carrying two copies of the long allele (activity-impulsivity scale of Dog-ADHD RS: p = 0.007; AIBS: p = 0.023). The results have some potential to support human studies; however, further research should reveal the molecular function of the TH gene variants, and look for the effect in more breeds.

## Introduction

Canine and human behaviour were shaped by similar evolutionary processes, therefore dogs demonstrate a complex level of similarity with humans in a set of functionally shared behavioural features (reviewed in [1]). Accordingly, due to a mixture of both homologies and analogies at different levels of biological organisations like genes and behaviour traits, dogs could serve as a useful model for studying the genetic background of complex human behavioural diseases [2]–[7]. However, the identification of the behavioural phenotype is often difficult. A widely-used method involves relying on breed stereotypes provided by experts such as dog-trainers [8], [9], but to reveal a valid association between behaviour and genetic factors, direct and precise behavioural phenotyping at the individual level is definitely as important as accurate genotyping.

Earlier Vas et al. [10] successfully adapted a human parental ADHD questionnaire [11] for dogs. The Dog-ADHD Rating Scale (Dog-ADHD RS) showed satisfactory test-retest and inter-observer reliability, internal consistency, and external validity. This finding was recently replicated on a large, predominantly North American sample [12].

Activity refers to self-initiated movement (e.g. [13]). This feature of behaviour is usually determined by measuring the ambulation of a dog in a closed arena with gridline on the floor (“open field”; e.g. [14]). According to Gosling and John [15] level of activity has links with the Extraversion dimension of the most widely accepted map of human personality structure: the Five Factor Model (FFM).

Human impulsivity is considered as the opposite of Conscientiousness, which includes facets as self-discipline, dutifulness and impulse control [15]. In dogs these facets are related to the Responsiveness to Training trait [16], assessed by for example retrieving an object [17] and by examining the dogs' reactions and interest in its environment across a variety of situations [18]. According to our knowledge, impulsivity was not tested directly on dogs by behavioural tests.

In this study we aimed at developing a valid and reliable test battery for measuring activity and impulsivity traits in dogs. Our second goal was to identify one of the underlying genetic factors of these complex traits.

Earlier, by using the Dog-ADHD RS [10] we found that police German Shepherd Dogs (GSDs) possessing at least one 3a allele in dopamine D4 receptor (DRD4) exon 3 showed significantly higher scores on the activity-impulsivity scale than dogs lacking this allele [19]. Subsequently, we found that repeat polymorphisms at exon 3 and intron 2 of the DRD4 gene contributed to the social impulsivity of pet GSDs [20]. Social impulsivity is manifested in approaching and following behaviour while encountering a friendly, unfamiliar experimenter.

Tyrosine hydroxylase (TH) is catalyzing the conversion of the precursor of dopamine (dihydroxyphenylalanine, DOPA). Dopamine is the precursor of the catecholamines norepinephrine (noradrenaline) and epinephrine (adrenaline). Dopamine is involved in the brain's reward system, and has many other functions in cognition, movement control, and attention [21]. Norepinephrine as a neurotransmitter plays a role in attention and focus and together with dopamine, they may be implicated in ADHD [22]. However, TH is found not only in the brain but in all cells containing catecholamines (“fight-or-flight” hormones), too.

The human TH gene is associated with mood disorders [23]–[24], personality factors [25] and may be involved in hypertension [26].

There is a high homology between the dog and human TH gene, and significant variations of the allelic frequencies among dog breeds suggest that the polymorphism of this gene is probably an important marker for the genetic background of behavioural characteristics in dogs [27]. Based on the metabolic pathways of TH, we suppose that it could be involved in the activity-impulsivity trait of dogs.

For the present study we developed a new test battery (Activity-Impulsivity Behavioural Scale, AIBS) for measuring canine activity-impulsivity. We investigated whether the activity-impulsivity trait in dogs measured by owners' report (Dog-ADHD RS [10]) and behaviour tests are affected by a recently reported TH intron 4 repeat polymorphism [28]. In this repeat polymorphism a 36-bp-long sequence in the intron 4 region of the TH gene is reiterated once (duplicated) or not reiterated at all. In case of the reiterated form of the microsatellite it consists of 334 bp (‘allele 2’), the single copy form spans 298 basepairs (‘allele 1’). The frequency of alleles varies among breeds, at least within the four breeds and the gray wolf (*Canis lupus*) investigated up to now. For example, the frequency of allele 2 is 31% in Groenandaels, 0.89% in German shepherds, and 0.73% in wolves. The polymorphic region is 92 basepairs away from the 5′ end of the intron. The biological function of the repeat variation is supposed to be the modulation of the splicing, as the polymorphism strongly affects the size of the intron. Moreover, based on the localization both the 5′ point and the branch point might be affected, thus the efficiency of the splicing of the two different variants is probably different.

The analysis was carried out within a single breed, the German Shepherd Dog. This breed is popular both as working (guide dog for the blinds, police dog, guarding and protection) and as pet dog. Regarding pet German Shepherds, a recent comparison of Hungarian and American (USA) dog behaviour and dog-keeping practices is also available, suggesting that activity-impulsivity scale of the Dog-ADHD RS is probably not associated with country, therefore the results could be generalized [29]. Age, sex and the level of training was also considered during the analysis as these variables may affect activity-impulsivity [10].

## Methods

### Ethics statement

No special permission for use of dogs in such non-invasive studies is required in Hungary. The relevant committee that allow to conduct research without special permissions regarding animals is: University Institutional Animal Care and Use Committee. (UIACUC, Eötvös Loránd University, Hungary). Owners attending dog training schools or responding to our advertisement at the department's homepage (http://kutyaetologia.elte.hu) volunteered to participate. Genetic analyses and behavioural testing of the animals were approved by the owners. The buccal smears were collected by the experimenters: Judit Vas, Ildikó Brúder, Borbála Turcsán and Enikő Kubinyi.

The persons shown in the photo gave written consent to the publication of the photo.

### Subjects

104 German Shepherd Dogs were involved in the study. Fifty-six dogs were males, 48 females; age range: 1–13 years, mean age: 3.92 years, SD = 2.7. 6% of male and 32% of female dogs were neutered. 58% of owners were women, 42% men; age range: 16–57 years, mean age: 32.7 years, SD = 11.8. 16.3% of the dogs had no formal training, 13.5% had basic training, 52.9% had one specialized training (e.g. rescue, agility), 17.3% had at least two specialized training. None of the subjects were closely related, i.e. littermate and parent-offspring relationships were excluded. Nine owners had two dogs in the sample. Owners attending dog training schools or responding to our advertisement at the department's homepage (http://kutyaetologia.elte.hu) volunteered to participate. Genetic analyses and behavioural testing of the animals were approved by the owners.

### DNA sampling and genotyping

Buccal smears were collected, and DNA was isolated with the Gentra purification kit (Valencia, CA). Repeat polymorphisms in the TH intron 4 were analyzed according to the procedure previously described in [28]. Shortly: The PCR reaction mixture contained 1 µM of each primer, approximately 5 ng of DNA template, 200 µM dATP, dCTP, dTTP, 100 µM of dGTP and dITP, 0.025 U HotStarTaq DNA polymerase 1x buffer and 1x Q-solution supplied by the Qiagen HotStarTaq polymerase kit in a 10 µl final volume. Conditions of the PCR cycle and the separation of PCR products by gel electrophoreses were as described before [28].

### Phenotyping

The behaviour of dogs were characterised by two instruments: Dog-ADHD Rating Scale [10], and the Activity-Impulsivity Behavioural Scale (AIBS).

#### I. Dog-ADHD Rating Scale (Dog-ADHD RS)

The Dog-ADHD RS was completed by the owner before the tests, in the presence of the experimenter. The questionnaire consists of two subscales. Seven items compose the activity-impulsivity scale (for example: ‘Your dog fidgets all the time’) and six items make up the inattention scale (for example: ‘It is difficult for your dog to concentrate on a task or play’). The scale scores were calculated for each dog as the mean of the scores given by the owner on a 4 point scale (from 0: never to 3: very often) [10].

#### II. Activity-impulsivity Behavioural Scale (AIBS)

Dogs were observed in a test battery conducted outdoors, on a remote area of a dog training school or at a quiet location on the campus of the Eötvös Loránd University 50 m away from the nearest building. The owner, a female experimenter and a camera-woman were present. The experimenter assessed the behaviour of the dogs at the testing location by filling in a score-sheet. The test-retest reliability of the AIBS was measured by retesting 14 dogs and comparing the test and retest AIBS scores. Retesting has been applied in a 1 week long dog camp. Interval between test and retest varied between 2 and 6 days. Owners were randomly asked to participate in the retesting. Inter-observer reliability was assessed by comparing the AIBS scores of the experimenter who scored the behaviour at the testing location, and of an independent coder, a trained biology MSc student, assessing 28 dogs from the video-recordings.

### Testing protocol (Figure 1)

**Figure 1:**
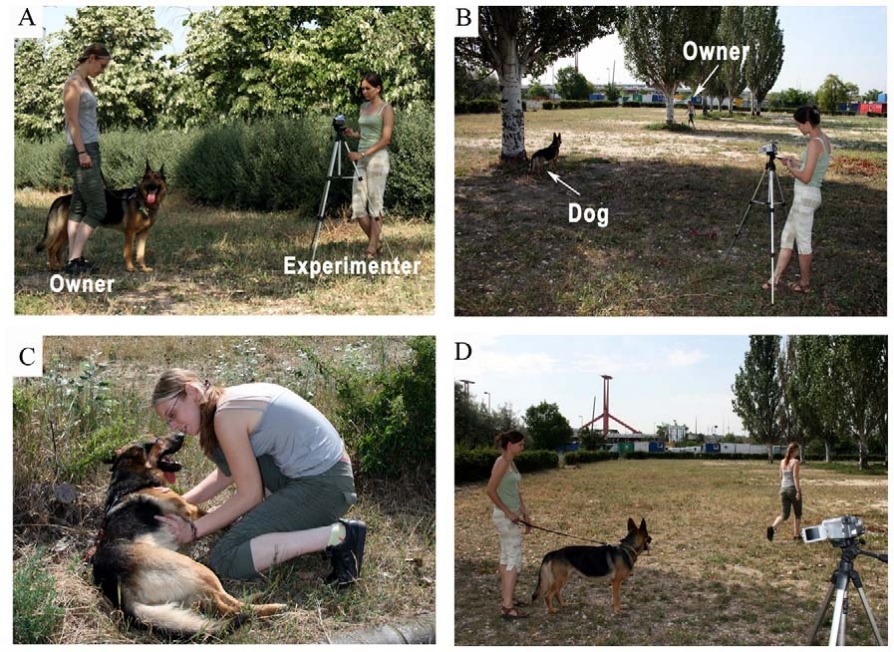
Illustrations for the subtests. A) Spontaneous activity: The owner stands still while holding the dog on a leash. B) Separation and play: The dog is tethered to a tree, while the owner is hiding behind a tree. C) Lying on the side: The owner commands the dog to lie down. Then owner crouches, turns the dog on the side and keeps the dog in this position for 30 seconds. D) Approaching the owner: The experimenter holds the dog on the leash, meanwhile the owner hides behind a large tree. After 30 seconds, the experimenter releases the dog.

The order of sub-tests was fixed.

#### 1. Spontaneous activity

The owner (O) stands still without paying special attention to the dog, while holding the dog on a leash (1.5-2 m). The dog is allowed to move freely within the range of the stretched leash and is not corrected or rewarded for any behaviour. This test lasts for 1 minute. Experimenter (E) stays at a distance of at least 3 m from the dog without paying any attention to the dog.

#### 2. Separation and play

The dog is tethered to a tree on a 3 m leash, while the O is hiding behind an object (e.g. a tree) 5-6 m from the dog, which blocks the dog from seeing the O. After 1 min has elapsed the E approaches the dog and initiates play with a tug (tug of war) for 30 s, and at the end she steps back to the camera. After 1 minute elapses E asks the O to come back.

#### 3. Lying on the side

O commands the dog to lie down. Then O crouches (down next to the dog) and turns the dog on the dog's side. O tries to keep the dog in this position for 30 seconds. If the dog gets up before the 30 seconds elapses, then the test restarts. Petting is allowed. If the dog refuses to lie on the side, or gets up again during the second try, the test is terminated after 60 seconds.

#### 4. Approaching the owner

E holds the dog on leash, and O is asked to hide behind a large tree or a house 15-20 m away from the dog. After 30 seconds, E releases the dog and says ”Go!”. If the dog does not start to move at once E gently by touches the rear end. If the dog does not approach the owner within 5 seconds, the E asks O to call the dog.

### Behavioural variables

All variables were coded on a 0-3 scale.

*Duration of moving the legs during the (1) Spontaneous activity test, (2) before the experimenter plays with the dog during the Separation and play test (1 min), and (3) after the experimenter plays with the dog during the Separation and play test (1 min)*: (0) no moving, (1) less than half of the time, (2) more than half of the time, (3) continuously.

(4) *Latency of lying down in the Lying on the side test:* (0) immediate lying down, (1) 1-14 s, (2) 15-30 s, (3) if the owner could not make the dog lie down on the side.

(5) *Duration of vocalization in the Approaching the owner test:* (0) no vocalization, (1) less than half of the time, (2) more than half of the time, (3) continuously.

(6) *Latency to approach the owner in the Approaching the owner test:* (0) no approach, (1) approach after calling, (2) approach in less than 5 sec, (3) immediate approach.

### Statistical analysis

SPSS for Windows was used for all statistical analyses. Cronbach's alpha was calculated to assess the internal consistency of the AIBS variables. Inter-observer and test-retest reliability was computed using intraclass correlation coefficient (ICC 1,1, one-way random single measures). Pearson correlation test was applied for investigating the links between the Dog-ADHD RS and AIBS. Multivariate General Linear model (MANCOVA) tested the main effects of independent variables (age, sex, training, TH genotype) on the Dog-ADHD RS and AIBS. Age was included as covariate, sex, training and TH genotype were fixed factors. Non-significant effects were removed through backward elimination. ANOVA with SNK post hoc test was used to investigate differences associated with trainings.

## Results

### Validity and reliability of the AIBS

The Cronbach's alpha was 0.68, satisfactorily high for the six behavioural variables. The mean scores of the Dog-ADHD RS subscales and the AIBS behavioural variables are presented in Table 1 and Table 2. For further analysis a scale score was calculated for each dog as the mean of the six variable scores.

**Table 1:** Mean scores of the Dog-ADHD RS subscales.

*Subscales*	*Mean score (SD)*
Activity-impulsivity	1.13 (0.59)
Inattention	0.91 (0.46)

**Table 2:** Mean scores of the AIBS behavioural variables.

*Variables*	*Mean score (SD)*
Duration of moving the legs in ‘Spontaneous activity’	1.47 (1.03)
Duration of moving the legs *before* the exp. plays with the dog in ‘Separation and play’	1.13 (0.81)
Duration of moving the legs *after* the exp. plays with the dog in ‘Separation and play’	0.89 (0.80)
Latency of lying down in ‘Lying on the side’	0.57 (0.95)
Duration of vocalization in ‘Approaching the owner’	0.64 (1.02)
Latency to approach in ‘Approaching the owner’	2.64 (0.75)

ICCs between two independent coders (inter-observer reliability) was 0.70.

Test-retest reliability was 0.79. Both scales of the Dog ADHD-RS correlated with the AIBS (activity-impulsivity: Pearson r = 0.53, p<0.001; inattention: Pearson r = 0.24, p<0.05), which means they assess the same construct (convergent validity). However, the correlation between AIBS and activity-impulsivity was significantly higher (z = −3.29, p<0.001).

### Genotype and allele frequencies

Two alleles were present in the German Shepherd Dogs (as reported previously [28]). In the short allele (allele 1) a 36-bp-long sequence is present as a single copy. In the long allele (allele 2) the sequence is in a duplicated form.

The genotype frequency did not deviate from the Hardy-Weinberg equilibrium (χ^2^_1_ = 1.14, p = 0.29). Two dogs (1.9%) were homozygotes for the short allele (1/1 genotype), 35 (33.7%) dogs were heterozygotes (1/2 genotype) and 67 dogs (64.4%) possessed the longer alleles exclusively (2/2 genotype). As the short allele was rare in the population, homozygotes (1/1) and heterozygotes (1/2) were combined for statistical analysis and defined as the genotype group containing individuals possessing at least one short allele.

### Effect of independent variables on the scales

After removing the non-significant effects through backward elimination, we found that TH and training status remained as explanatory variables in the model (TH: F_3,97_ = 3.002, p = 0.034; training: F_3,97_ = 2.065, p = 0.033).

**Figure 2:**
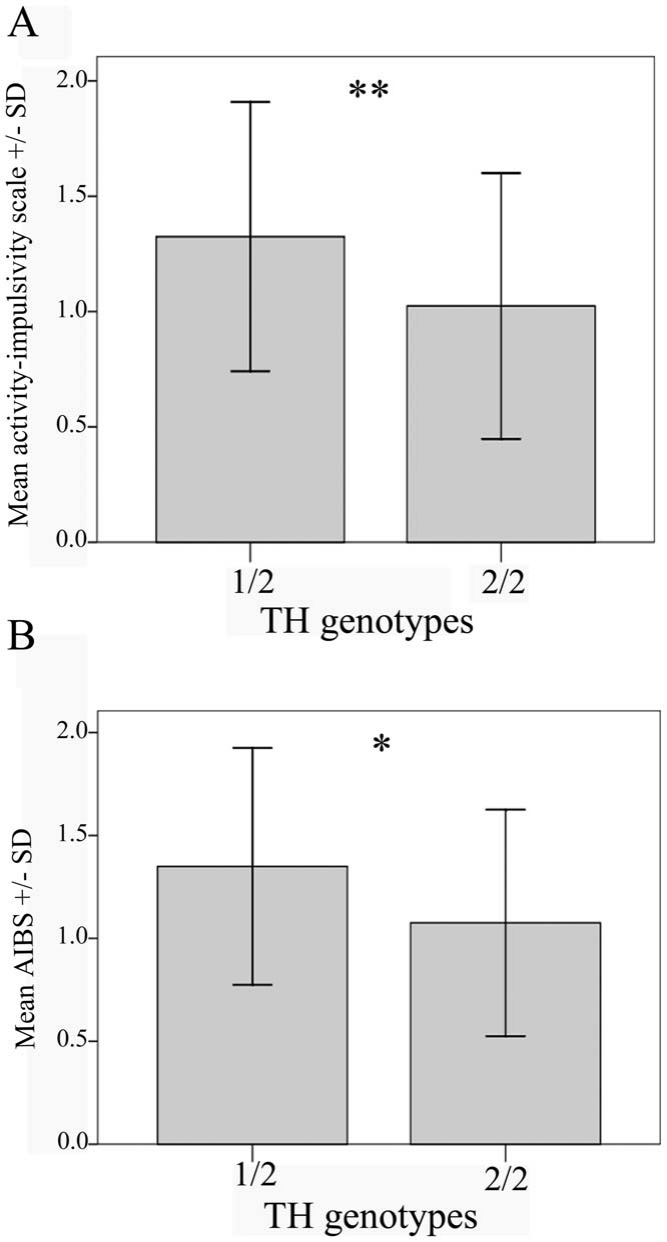
Association between tyrosine hydroxylase intron 4 genotypes and activity-impulsivity related phenotypes. (A) Dog-ADHD RS activity-impulsivity scale; (B) Activity-Impulsivity Behavioural Scale (AIBS). 1/2 genotype represents the group of individuals possessing at least one short allele. Subjects in the 2/2 genotype possess two long alleles. Box-plot figures with sample minimum and maximum, lower and upper quartiles and medians. * indicates significant differences (* p<0.05; ** p<0.01).

The TH polymorphism was associated with both the Dog-ADHD RS activity-impulsivity scale (F_1,99_ = 7.489, p = 0.007), and the AIBS (F_1,99_ = 5.299, p = 0.023). The genotype group containing dogs possessing at least one short allele was reported to be more active-impulsive by the owners and reached higher scores on the behavioural scale than homozygotes possessing the longer alleles exclusively (Figure 2). Omitting the rare 1/1 genotype (two dogs) did not affect the results significantly.

The training status had effect on the inattention scale only (F_3,97_ = 5.136, p = 0.002). Dogs with one or two specialized training were reported by the owners to be less inattentive than individuals with basic training or not trained at all.

## Discussion

The aim of this study was twofold. First, we demonstrated internal consistency, inter-observer reliability, test-retest reliability, and convergent validity of the Activity-impulsivity Behavioural Scale (AIBS), and investigated its links with the age, sex and training status of dogs.

High scores in the test were characterized by high motor activity, high latency of lying down on the side, high amount of vocalization during separation from the owner and fast approach to the hiding owner. Importantly, test-retest and inter-observer reliability indicated that the behavioural scoring system is reliably applicable at the experimental place. This effectively decreases the time needed for behavioural evaluation (i.e. the experimenter at the spot scored the dogs similarly as an independent observer did so during watching the video-recordings).

The existence of a reliable and valid test battery is crucial for measuring consistent traits [30]. Noteworthy, that the convergent validity, which was assessed by the correlation coefficient between the owner report (Dog-ADHD RS) and behaviour scores (AIBS), was higher (r = 0.54) compared to the mean correlation (r = 0.27) between personality judgements (for the Extraversion, Agreeableness, Neuroticism and Openness dimensions) and behaviour ratings of dogs in a previous study [31].

In previous research assessing a trait, questionnaire rating and behaviour coding or scoring have usually been made by the same individual, thereby compromising the independence of the two measures (but see [32]). In the present study different individuals were used as raters and coders. Coders were completely unacquainted with the subjects, while raters were the owners of the dogs. However, it is known that owners can be biased in their views of their dogs, and their ratings may be vulnerable to appearance-based stereotypes [33]. We tried to avoid this bias by using a single breed. Moreover, recent research has suggested that different levels of experience with dogs are not critical in rating the majority of behaviours ([28], [30], [31]).

Age and sex of the dogs had no effects on the scales of the Dog-ADHD RS in this study. However, training strongly affected the inattention of dogs. Highly trained dogs were reported to be less inattentive. Interestingly, activity-impulsivity trait was not affected by training. Dogs with a specialized knowledge (rescue, agility, guarding, etc) were reported and scored similarly active-impulsive as not or basic trained dogs. This suggests that activity-impulsivity does not counteract with performance during typical tasks in the GSD breed.

Our second aim was to search for associations with a genetic factor, TH polymorphism. We found that TH intron 4 polymorphism was associated with activity-impulsivity trait measured by both the Dog ADHD RS (owner report) and the AIBS (behavioural scoring). Dogs possessing at least one short allele were reported and found to be more active-impulsive, compared to animals possessing the long allele exclusively. This effect was independent of age, sex and training status of the dogs.

According to our knowledge, this is the first report of a TH-behaviour trait association in dogs. The result is in harmony with an earlier assumption that general activity might be related to the TH gene polymorphism in dogs [27]. However, we should note that the former study involved breed comparisons. Although breed comparisons could be thought-provoking, such results do not exclude the possibility that the effect is not linked to the target gene but to other breed-specific genetic or environmental factors. Within-breed phenotype-gene associations always provide stronger evidence because quantitative association studies have small effect sizes, and the characteristic genetic constitutions of the breeds could overshadow the slight effect of candidate genes. Furthermore, in contrast to [27], where four SNPs were identified, our study investigated a repeat polymorphism of the TH gene, which could be analogous with that of humans. The human TH gene contains an informative tetranucleotide repeat in 5-11 copies within intron A, which may function as a transcriptional enhancer, thus it may be directly involved in the transcriptional regulation of the TH gene [25]. This microsatellite was found to be associated with neuroticism and extraversion [27] [36], which are linked with facets of impulsivity in humans [37]. Thus our result (association between repeat polymorphism in intron of the TH gene and the activity-impulsivity trait in dogs) is in accordance with human studies. However, association studies do not provide exclusive evidence, and a replication study is required for any definite conclusion for TH effect on dog activity-impulsivity.

Our finding suggests that the dog model might help to understand the underlying genetic factors of complex traits in humans. However, further study is needed to reveal the molecular function of the TH gene variants, look for the effect in more dog breeds, and find out how activity-impulsivity trait mimics human ADHD features.

### Conclusions

In this study we have investigated whether there is an association of the tyrosine hydroxylase gene with activity-impulsivity and inattention trait in a pet German Shepherd Dog population, assessed by dog owners filling in a questionnaire, and by experts scoring the behaviour of dogs performing in a test battery. We have found, that the tyrosine hydroxylase intron 4 repeat polymorphism was related to both the questionnaire and the behavioural test scale. To our knowledge, applying multiple instruments to measure a trait for detecting gene-behaviour association in animals is unique in the literature. Previous studies used either rating of traits (e.g. horse: [38], dog [39], [40] or behavioural coding exclusively (e.g. great tit: [41], rhesus macaque: [42], vervet monkey [43]).

Moreover, our study reveals new information about a popular working and pet dog breed, and could provide novel means for diagnosing canine hyperactivity. The present results also have some potential to support human studies; however, further studies should examine other personality traits involved in the activity-impulsivity of dogs, and the links with human ADHD.
